# Non‐Invasive Photoacoustic Cerebrovascular Monitoring of Early‐Stage Ischemic Strokes In Vivo

**DOI:** 10.1002/advs.202409361

**Published:** 2024-12-04

**Authors:** Jiwoong Kim, Joo Young Kweon, Seongwook Choi, Hyunseo Jeon, Minsik Sung, Rongkang Gao, Chengbo Liu, Chulhong Kim, Yong Joo Ahn

**Affiliations:** ^1^ Departments of Convergence IT Engineering Medical Science and Engineering Electrical Engineering and Mechanical Engineering Pohang University of Science and Technology (POSTECH) Cheongam‐ro 77, Nam‐gu Pohang Gyeongbuk 37673 Republic of Korea; ^2^ Research Center for Biomedical Optics and Molecular Imaging Key Laboratory of Biomedical Imaging Science and Systems Shenzhen Institute of Advanced Technology Chinese Academy of Sciences Shenzhen 518055 China; ^3^ Opticho Inc. Pohang Gyeongbuk 37673 Republic of Korea; ^4^ Institute for Convergence Research and Education in Advanced Technology Yonsei University Yonsei‐ro 50, Seodaemun‐gu Seoul 03722 Republic of Korea

**Keywords:** brain imaging, ischemic stroke, multiparametric imaging, photoacoustic imaging

## Abstract

Early‐stage stroke monitoring enables timely intervention that is crucial to minimizing neuronal damage and increasing the extent of recovery. By monitoring collateral circulation and neovascularization after ischemic stroke, the natural recovery process can be better understood, optimize further treatment strategies, and improve the prognosis. Photoacoustic computed tomography (PACT), a non‐invasive imaging modality that captures multiparametric high‐resolution images of vessel structures, is well suited for evaluating cerebrovascular structures and their function. Here 3D multiparametric transcranial PACT is implemented to monitor the early stage of a photothrombotic (PT)‐stroke model in living rats. New vessels in the PT‐induced region are successfully observed using PACT, and these observations are confirmed by histology. Then, using multiparametric PACT, it is found that the SO_2_ in the ischemic area decreases while the SO_2_ in newly formed vessels increases, and the SO_2_ in the PT region also recovers. These findings demonstrate PACT's remarkable ability to image and monitor cerebrovascular morphologic and physiological changes. They highlight the usefulness of whole‐brain PACT as a potentially powerful tool for early diagnosis and therapeutic decision‐making in treating ischemic stroke.

## Graphical abstract

Combining raster and rotational scanning, this study presents 3‐D whole‐brain photoacoustic computed tomography (PACT) of the rat brain. Angiogram and oxygen saturation distribution images are captured at whole‐brain scale, applying multiparametric analysis to photothrombotic strokes, and cerebrovascular changes are observed at 1 day after stroke induction.

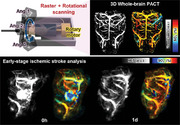

## 1 Introduction

Stroke, resulting from an acute blockage of cerebral blood flow, is the second leading cause of mortality worldwide.^[^ ^1^ ^]^ Approximately 85% of stroke cases are ischemic strokes, which lead to rapid neuronal death from insufficient blood supply.^[^ ^2^ ^]^ Thrombolysis and thrombectomy are the two main treatments for ischemic stroke.^[^ ^3^ ^]^ Thrombolysis is an emergency treatment that uses medications such as tissue plasminogen activator to restore blood flow by dissolving blood clots. It is most effective within the first 3 to 4.5 h and becomes less effective over time.^[^ ^4^ ^]^ Thrombectomy, the endovascular mechanical removal of the clot, can be performed within 6 to 24 h of stroke onset.^[^ ^5^ ^]^ Because the window for treating a stroke is generally only a few hours, a rapid and accurate diagnosis is required to increase the patient's chances of recovery and to reduce the mortality rate. Therefore, identifying key indicators (e.g., the detailed anatomical location of the ischemic region, the degree of cerebrovascular obstruction, cerebral oxygen saturation (SO_2_) levels, and the potential for hemorrhagic transformation) is paramount for the early diagnosis of ischemic stroke. Of particular interest, collateral circulation and neovascularization can provide an alternative blood flow to the ischemic region, potentially limiting neuronal damage and improving recovery.^[^ ^6^, ^7^ ^]^ Monitoring these cerebrovascular changes is crucial for understanding the extent of these natural compensatory mechanisms, and it can guide further therapeutic decisions to enhance them.^[^ ^8^ ^]^

X‐ray computed tomography (X‐ray CT) and magnetic resonance imaging (MRI) are conventionally employed in the early detection and assessment of ischemic stroke. These techniques provide valuable insights into the structural changes (e.g., vessel obstruction, collateral circulation, vessel formation) induced by stroke.^[^ ^9^, ^10^, ^11^, ^12^ ^]^ However, these conventional modalities have certain limitations. X‐ray CT involves exposure to ionizing radiation and requires a contrast agent to image blood vessels. MRI, although providing superior soft tissue contrast and precise structure images, may have insufficient temporal resolution to identify early‐stage ischemia alterations.^[^ ^13^ ^]^

Photoacoustic imaging (PAI), which is both non‐invasive and non‐ionizing, has significant advantages over conventional methods for early‐stage monitoring of ischemic stroke.^[^ ^13^, ^14^, ^15^, ^16^, ^17^, ^18^ ^]^ PAI provides high‐resolution images along with multiparametric information about biological tissues based on the absorption of pulsed laser light and subsequent emission of ultrasonic waves, known as the photoacoustic (PA) effect.^[^ ^19^, ^20^, ^21^, ^22^ ^]^ The unique light absorption characteristics of biomolecules enable PAI to non‐invasively observe anatomical structures and physiological functions.^[^ ^23^, ^24^, ^25^, ^26^, ^27^, ^28^, ^29^ ^]^ PAI can be implemented as either photoacoustic microscopy (PAM) or photoacoustic computed tomography (PACT). PAM typically uses a single transducer to acquire PA signals, achieving high‐resolution images of relatively superficial tissues.^[^ ^20^, ^30^, ^31^, ^32^, ^33^, ^34^, ^35^ ^]^ Despite its restricted field‐of‐view (FOV) and relatively shallow penetration depth, this modality provides fine details of the microvasculature and cellular levels.^[^ ^20^, ^36^, ^37^, ^38^, ^39^, ^40^ ^]^ In comparison, PACT can image deeper structures over a larger FOV, which is crucial for monitoring internal organs and cerebral structures. PACT reconstructs 3D volumetric images by using multiple transducer elements to simultaneously detect PA signals, providing preclinical research with valuably intuitive images.^[^ ^41^, ^42^, ^43^, ^44^, ^45^ ^]^ Based on these advantages, PACT can be successfully employed in ischemic stroke studies, as it can capture both the structural and functional dynamics within the brain, including angiogenesis and SO_2_ changes.^[^ ^23^, ^24^, ^25^, ^46^, ^47^, ^48^ ^]^ With high temporal resolution and a large FOV, PACT facilitates immediate assessment and continuous monitoring of the pathophysiological changes following ischemic stroke.

In studies of middle cerebral artery occlusion (MCAO)‐ and photothrombotic (PT)‐induced stroke models, the stroke infarction region has been identified through PACT.^[^ ^13^, ^16^, ^49^, ^50^, ^51^, ^52^ ^]^ PACT revealed lower blood perfusion and SO2 values in the infarct region than in the non‐infarct region. However, in previous studies, blood vessels could not be visualized in high definition throughout the whole brain due to limited field‐of‐views (FOVs) that impeded monitoring likely cerebrovascular changes following by vessel occlusion. Cerebrovascular changes in early‐stage ischemic stroke, such as collateral circulation, which occurs throughout the entire cerebral vasculature, and they play crucial roles in maintaining blood flow during ischemia, limiting tissue damage and improving recovery outcomes.^[^ ^53^ ^]^ Since this circulation spans the entire brain, it is beneficial to achieve whole brain imaging to comprehensively assess these events.^[^ ^54^ ^]^ In this study, we present a whole‐brain PACT system that overcomes the limited FOV, employing a hemispherical transducer array and a partial‐rotary‐scanning module to monitor early ischemic stroke through high‐definition morphologic and functional imaging of the whole‐brain vasculature.^[^ ^55^, ^56^ ^]^ A hemispherical transducer array is recognized as the most nearly optimal detector for PACT, simultaneously acquiring omnidirectional PA signals with minimized limited‐view effects.^[^ ^41^, ^57^ ^]^ By employing a hemispherical transducer array with partially rotary scanning, we were able to achieve whole‐brain angiograms and capture cerebrovascular changes across the entire brain, enabling the first observations of neovascularization and collateral circulation during early‐stage ischemic stroke (see Table S1, Supporting Information for a summary of similar studies). We also captured vessel‐specific SO_2_ changes in the brain, observing hypoxia in the thrombosis and increased SO_2_ in the collateral circulation that developed to maintain the oxygen supply in the PT‐induced ischemic stroke model. Through this study, we demonstrated that the high‐definition whole‐brain PACT system can image early‐stage ischemic stroke multiparametrically, and we expect it to play an important future role in early stroke diagnosis.

## 2 Results

### 2.1 Whole‐Brain PACT System and In Vivo Morphological Imaging

**Figure 1:**
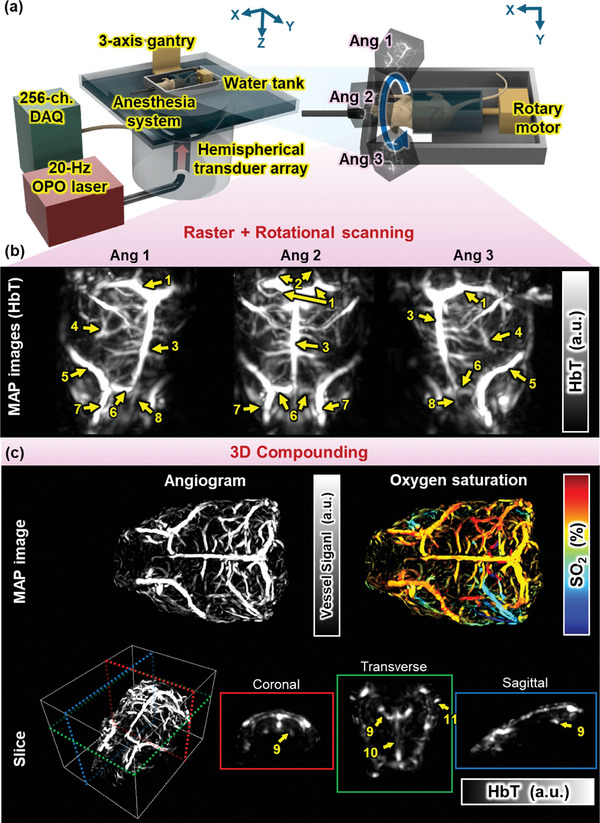
Whole‐brain photoacoustic computed tomography system and in vivo morphological imaging of rat whole brain. (a) Schematic of the whole system and rotational scanning module. DAQ, data acquisition board; OPO, optical parametric oscillator; (b) Maximum amplitude projection images of total hemoglobin volume acquired from each of three angles in the rotational system. Ang, Angle of the head. (c) 3D whole‐brain image. HbT, total hemoglobin; SO_2_, oxygen saturation; 1, transverse sinus; 2, sigmoid sinus; 3, superior sagittal sinus; 4, middle cerebral artery; 5, rostral rhinal vein; 6, inferior cerebral vein; 7, supraorbital vein; 8, olfactory sinus; 9, interpterygoid emissary vein; 10, cavernous sinus; and 11, retroglenoid vein.

The whole‐brain PACT system (**Figure** **1**a) is composed of a 1024‐element hemispherical transducer array, a 20‐Hz tunable optical parametric oscillator (OPO) laser, and a 256‐channel data acquisition board (DAQ) system. Light is delivered through a customized fiber bundle inserted in the center of the transducer. Two distinct configurations of motorized scanners are employed: The first configuration, using a tri‐axial gantry stage, enabling raster scanning in the XY‐plane of a rat that is partially submerged in a water tank. The other configuration, using a custom‐built rotational scanning module, stepwisely rotates the rat body to image three angles of the rat brain (Figure 1a). By rotating the rat brain through this rotational scanning, the entire brain was more comprehensively imaged. We acquired a 3D whole‐brain PACT image by compounding three volumes (see Experimental Section for further details of the imaging protocol). To acquire total hemoglobin and oxygen saturation information through spectral‐unmixing, the rat brain is imaged at six wavelengths (756, 780, 796, 820, 866, and 900 nm). To validate the SO_2_ measurements from the system, we measure the SO_2_ in a human forearm (Figure S1, Supporting Information see Experimental Section). The results are consistent with the well‐known ranges of 83.3% ± 0.7% for the radial artery and 60.4% ± 0.7% for the radial vein.^[^ ^58^, ^59^ ^]^ With this validated system, we utilized the simultaneous acquisition of the vascular structures and the SO_2_ information of whole‐brain volume to analyze early‐stage ischemic stroke intuitively. Additionally, we conducted *ex vivo* skull sample experiments to evaluate the brain imaging performance of the PACT system.^[^ ^60^ ^]^ Using nylon threads which have a diameter of 160–180 µm as targets, we observed a resolution of 390 µm without the skull,^[^ ^55^ ^]^ but a reduced resolution of 450 µm with the skull (Figure S2, Supporting Information).

PACT‐brain images obtain at three angles in a rotary scan, shown in Figure 1b, clearly show the major cerebral vessels, indicated with arrows and labeled respectively as follows: 1, the transverse sinus (TS); 2, the sigmoid sinus (SS); 3, the superior sagittal sinus (SSS); 4, the middle cerebral artery (MCA); 5, the rostral rhinal vein (RRhV); 6, the inferior cerebral vein (ICV); 7, the supraorbital vein (SV); and 8, the olfactory sinus (OS) (Figure 1b). By compounding the images from the three angles, a rat whole‐brain PACT image with a wide FOV can be obtained, as shown in Figure 1c and Figure S3 (Supporting Information) (See the Experimental Section). Figure 1c shows the maximum amplitude projection (MAP) image of the angiogram obtained by applying a vesselness filter to the acquired whole‐brain total hemoglobin (HbT) volume image, along with the corresponding SO_2_ distribution volume images (image processing details are in the Experimental Section, and Movie S1 (Supporting Information) shows 3D rendered images). As a side note, we observed that the SO_2_ levels of the lateral blood vessel regions on both sides are asymmetric, the result of strong compressive force to one side of the rat's head during fixation. In both the single‐angle‐ and whole‐brain PACT images, vessels in the middle part of the rat brain, such as the SS, SSS, and ICV, are clearly observed. However, vessels such as the TS and RRhV, which are only partially visible in the single‐angle‐brain image, are clearly visible in the whole‐brain image, and their connectivity is also clearly revealed, allowing the cerebral vessel network to be seen more accurately through the wide FOV of whole‐brain PACT imaging (Figure S3, Supporting Information). The B‐mode images in Figure 1c and Figure S3 (Supporting Information) are cross‐sections of the regions bounded by the dotted rectangles of each color in the 3D‐rendered image (see Movie S2, Supporting Information for B‐mode section images and Movie S3, Supporting Information for video of depth‐encoded images from skin surface). The difference between single‐angle and whole‐brain views can be confirmed for cross‐sections at the same location, which the whole‐brain PACT can observe deep brain anatomies (9, interpterygoid emissary vein (IPTGV); 10, cavernous sinus (CAV); and 11, retroglenoid vein (RGLV)). Notably, the parts marked with orange arrows in Figure S3 (Supporting Information) are clearly visible in the whole‐brain image, but cannot be confirmed in the single‐angle brain image.

### 2.2 Cerebrovascular PACT Monitoring of Photothrombotic‐Stroke Model In Vivo

**Figure 2:**
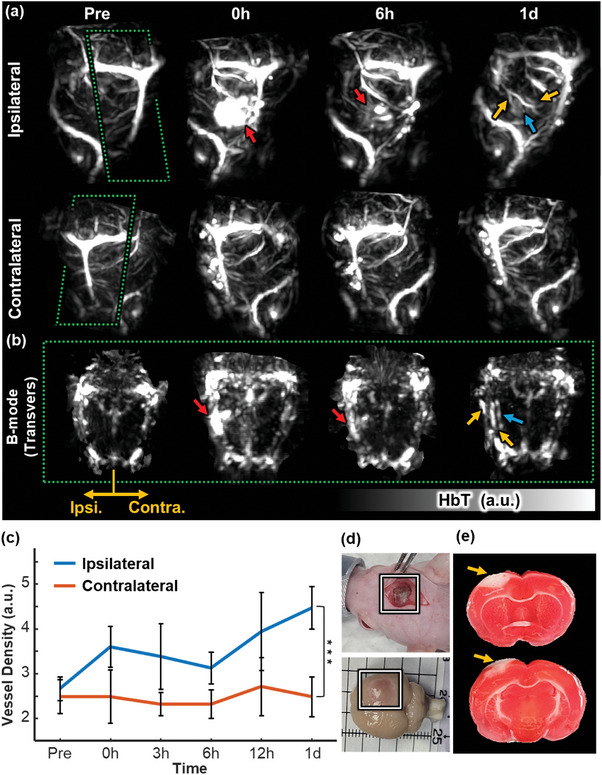
Morphologic analysis of the cerebrovascular system in early‐stage ischemic stroke. (a), Maximum amplitude projection images at pre‐, 0‐, and 6 h, and 1‐day after induced photothrombosis (b), B‐mode slice images of the rat brain at corresponding time points. (c), Vessel density changes after photothrombosis of the ipsilateral and contralateral sides of the brain. (d), Photographs of the rat brain immediately after photothrombotic stroke induction (top) and after dissection at the end of Day 1 (bottom). (e), Triphenyl tetrazolium chloride (TTC) staining results. ^***^ *p* < 0.005.

Utilizing the cerebral vascular imaging capabilities of the whole‐brain PACT, we monitor the morphologic changes following an induced PT‐stroke in the rat brain. The Methods describe the details of the PT‐stroke models. First, based on the HbT volume, we analyzed the blood vessel structures in the PT‐stroke model brain. In the analysis of the PT‐stroke model, the bulk structure of thrombosis can distort the visualization of the thrombosis structure when applying the vesselness filter. Therefore, we analyze based on the HbT volume before applying filter. (Note, later quantification of vessel density was performed using the angiogram excluding the thrombosis area, and the angiogram applying vesselness filter on Day 1 which thrombosis signals are weakened is shown in Movie S4, Supporting Information) **Figure** **2**a is a MAP image of HbT in the ipsilateral and contralateral regions. Immediately after PT induction (Hour 0), a strong signal is observed at the induction region on the ipsilateral side (red arrows), then the thrombosis signal decreases over time until Day 1 (blue arrow). Importantly, as the thrombosis signal resolves, new blood vessels are observed on Day 1 (yellow arrows in Figure 2a). In contrast, the contralateral hemisphere does not show any vascular changes (see Movie S5, Supporting Information for 3D rendered image at pre‐, 0‐, and 6 h, and 1‐day after induction). Additionally, Figure 2b shows clear differences in vascular structures between the contralateral and ipsilateral hemispheres in the transverse B‐mode slice images. (Same color of arrows in Figure 2b indicates the corresponding structure in Figure 2a) We quantitatively analyze (*n* = 7) the vessel density based on the vessel angiogram on the ipsilateral side and compare it with the contralateral side (Figure 2c, and details in the Experimental Section). The vessel density on the ipsilateral side increases from 2.66 ± 0.27 a.u. at pre‐induction to 4.47 ± 0.48 a.u. at Day 1, while the vessel density in the contralateral hemisphere remains essentially the same, ranging from 2.49 ± 0.38 to 2.49 ± 0.44 (*p* = 0.004 between the ipsilateral and contralateral hemispheres on Day 1). This Day 1 increase in vessel density in the ipsilateral hemisphere reflects cerebrovascular changes to maintain blood flow in the stroke‐induced region. Further, we confirm ischemic stroke lesions by photographing brains immediately after PT induction and brain dissection on Day 1 (Figure 2d), along with triphenyl tetrazolium chloride (TTC) staining to verify the stroke induction (Figure 2e). The regions of thrombosis and stroke are indicated by white boxes and yellow arrows. The newly observed vessels and the increase in vessel density in the ipsilateral region captured through PACT can be explained by venous collateral circulation and neovascularization. In acute ischemic stroke, vascular pressure increases due to the formation of a thrombus in the blood vessel, and venous collateralization occurs, which is a phenomenon in which the venous vessels that were clumped together are distended by the increased pressure of blood flowing toward the vein.^[^ ^54^, ^61^, ^62^ ^]^ In conclusion, we observed newly flowing blood vessels on Day 1 that were not detected before PT‐stroke, and also observed increased in the vessel density in the ipsilateral region, confirming the occurrence of neovascularization and collateral circulation in early‐stage ischemic stroke.

**Figure 3:**
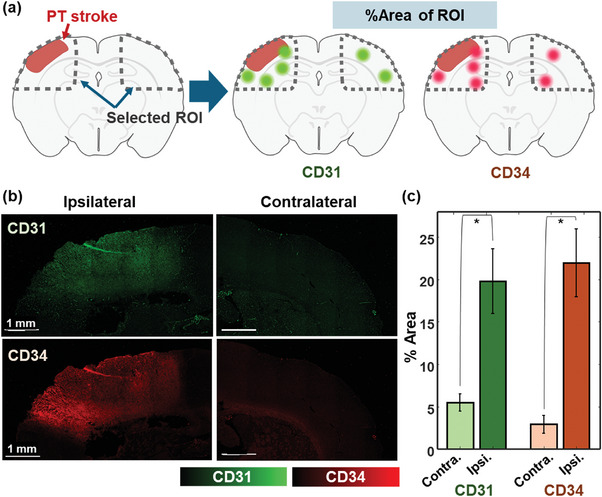
Histological analysis of a rat brain at Day 1 after PT‐stroke induction. (a) Schematic of the histological analysis. (b) CD31 and CD34 stained images from ipsilateral and contralateral hemispheres. (c) CD31 and CD34 stained area percentages in each hemisphere slice. Data points are indicated with crosses. ^*^ *p* < 0.05.

To validate these cerebrovascular changes observed through PACT imaging, we conduct histological analyses using CD31 and CD34 staining. **Figure** **3**a is a schematic of the histological validation process, comparing the percentages of the affected areas (%Area) in designated ROIs from each hemisphere (details in the Experimental Section). Figure 3b shows higher CD31 and CD34 intensities in the ipsilateral brain section than in the contralateral region of the corresponding rat brain in Figure 2, validating the observed neovascularization in the ipsilateral region (see Figure S4, Supporting Information for other ROI images). These findings are further confirmed through quantitative analysis (*n* = 4), with the results shown in Figure 3c. We assess the histologic difference by comparing the area ratio of values exceeding the threshold among all pixels on each side. The %Area of CD31 and CD34 stain in the ipsilateral region are 19.78 ± 3.82% and 21.97 ± 4.02%, respectively, showing several times higher signals than the values of 5.48 ± 1.02% and 2.93± 1.06% in the contralateral region (*p* = 0.02 in CD31, *p* = 0.01 in CD34, between the ipsilateral and contralateral regions). These histological analyses support the PACT imaging results, confirming neovascularization and collateral circulation to maintain blood supply in early‐stage ischemic stroke. The relatively high CD34 staining results strongly indicate neovascularization in the ipsilateral region. Also, the increase in vessel density observed in PACT‐based imaging, as well as in CD31 and CD34 staining, collectively confirm the presence of collateral circulation after PT‐stroke. In conclusion, we successfully demonstrate the cerebrovascular changes in the PT‐stroke‐induced region through PACT monitoring of the whole brain, including the ipsilateral and contralateral hemispheres.

### 2.3 In Vivo PACT Monitoring of the Oxygen Saturation of a Photothrombotic Stroke

**Figure 4:**
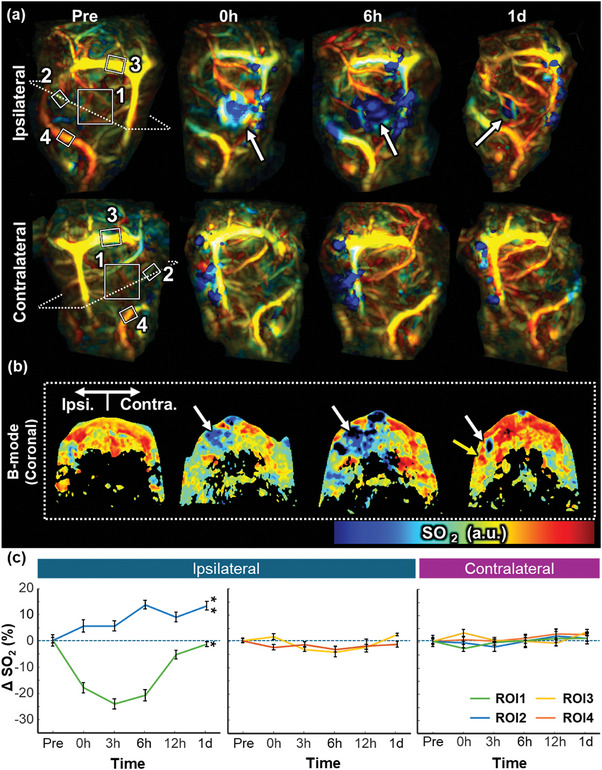
Oxygen saturation of early‐stage ischemia in a region with a photothrombotically induced stroke. (a) Maximum amplitude projection oxygen saturation distribution images at pre‐, 0‐, 6 h, and 1‐day after photothrombotic stroke induction (b) B‐mode slice images of the rat brain at corresponding time points (c) Vessel‐specific oxygen saturation changes in the ipsilateral and contralateral sides of the brain after photothrombosis. ROI 3 (*p* = 0.2) and ROI 4 (*p* = 0.4) did not show statistically significant differences between the ipsilateral and contralateral sides. ^*^ *p* < 0.05; ^**^ *p* < 0.01.

Capitalizing on PACT's ability to monitor oxygen saturation, we analyze the changes in cerebral SO_2_ following a PT‐induced stroke, using whole‐brain PACT (details in Experimental Section). **Figure** **4**a displays the SO_2_ distribution in the PT‐induced‐stroke brain region. Notably, the SO_2_ level is significantly lower in the PT region, where strong PA signals are detected in the structural images (white arrows). In addition, we observe high oxygen saturation levels in newly formed vessels on Day 1, where collateral circulation is seen in the morphologic analysis (Figure 2a). As with the morphologic images, no significant changes in SO_2_ are observed over time in the contralateral region (A comparable image from the control group, in which PT was not induced, can be found in Figure S5, Supporting Information). In Figure 4b, hypoxia (ischemic core, white arrows) and surrounding vessels with relatively high SO_2_ (yellow arrows) in the PT‐induced area are also confirmed through B‐mode slice images. We quantify (n = 7) the changes in SO_2_ of cerebral vessels by calculating the SO_2_ in four specific vessel regions, some of which correspond to the area where thrombosis is induced. The white boxes in Figure 4a,c show the quantitative changes in vessel‐specific SO_2_ after PT induction, representing difference of SO_2_ from pre‐induction time points. First, in the region where thrombosis is induced (ROI 1), the SO_2_ decreases to −26.0% ± 2.2% at 3 h after induction, but by Day 1 it has recovered to −1.5% ± 1.0%. In vessel ROI 2, where the newly formed thrombosis is resolved, the SO_2_ increases to 14.2% ± 1.7% from pre‐induction to Day 1. To compare the trend with the contralateral region, we designated the end region of the newly formed vessels also observed at the pre‐induction point is quantified. See white box number 2 in Figure 4a. On the other hand, the major cerebral vessels relatively maintaine SO_2_ values from pre‐induction time point (ROI 3: 2.4% ± 0.6% at Day 1; ROI 4: −1.4% ± 1.3% at Day 1). This observation contrasts with the analysis of the contralateral hemisphere without the stroke, where all these vessel regions show no significant changes in SO_2_ (ROI 1: 2.8% ± 0.7% at Day 1; ROI 2: 1.2% ± 2.3% at Day 1; ROI 3: 3.9% ± 0.8% at Day 1; ROI 4: 1.2% ± 1.2% at Day 1). There are significant differences in the ROI 1 and ROI 2 values between the ipsilateral and contralateral sides on Day 1 (*p* = 0.04 in ROI1, *p* = 0.005 in ROI2), indicating changes in SO_2_. However, the major cerebral vessels in the stroke‐induced region do not show statistically significant changes in the ipsilateral region on Day 1 (*p* = 0.2 in ROI3, *p* = 0.4 in ROI4), indicating these vessels maintained their SO_2_ levels. By analyzing vessel‐specific SO_2_ changes with PACT, we verify hypoxia in the PT region and increased SO_2_ levels in newly formed vessels.

## 3 Conclusion

In this study, we applied 3D whole‐brain PACT to monitor anatomical and SO_2_ changes in the cerebrovascular system of rats with an induced a PT‐stroke. Previous studies have used various imaging techniques to investigate the effect of induced ischemic stroke.^[^ ^14^, ^63^ ^]^ However, assessing early stroke accurately requires an imaging modality that can simultaneously provide adequate temporal resolution, a wide FOV, and multiparametric information. Traditional stroke research has employed TTC staining, laser speckle imaging, computed tomography (CT), and magnetic resonance imaging (MRI).^[^ ^9^, ^10^, ^11^, ^12^ ^]^ However, each of these methods has drawbacks. TTC staining can identify the lesion site only after sacrificing the animal, laser speckle imaging has low depth resolution, and CT and MRI require expensive equipment and long acquisition times. In comparison, the PACT system presented in this study avoids these shortcomings. PACT can monitor vascular changes with deep penetration depth in real‐time, and it offers multiparametric information, including SO_2_ changes. These compelling advantages make it an important tool for understanding overall cerebrovascular changes in the early stages of stroke.

Through high‐definition multiparametric imaging using the developed PACT system, we were able to observe vascular changes in vivo in the brains of rats with early‐stage ischemic stroke. We observed neovascularization and collateral circulation and were able to analyze the vessel density in the PT‐induced region. These observations were validated by histological analyses using CD31 and CD34 staining, confirming the changes in the ipsilateral region and corroborating the PACT results. Additionally, our study verified significant changes in SO_2_ levels in the PT‐induced stroke region. The decreased SO_2_ in the PT region and the higher SO_2_ levels in newly observed vessels after PT induction successfully verified cerebral oxygenation dynamics after ischemic injury. Monitoring SO_2_ changes in specific vessel regions provides valuable insight into early‐stage stroke and recovery processes in living rats. In addition, while previous stroke studies have observed neovascularization, there has been a relative lack of research on collateral circulation after stroke induction.^[^ ^64^ ^]^ Using the PACT system, we were able to observe the overall cerebrovascular reactivities following ischemic stroke, including collateral circulation. This imaging modality provides new insights into revascularization after stroke and will be an important tool for future research.

Despite these results, our study has some limitations. First, it was challenging to reproduce the imaged rat's initial posture accurately when fixing the rat brain in to the scanning module at each time point. This difficulty will be overcome through advanced hardware systems or image registration algorithms that enable further detailed morphologic analysis.^[^ ^65^ ^]^ Second, as confirmed by the ex vivo skull sample experiments, transcranial imaging is affected by the skull, and this issue can be improved with further research. Studies on aberration correction methods using image processing algorithms could provide a potential solution to minimize the effects of the skull on transcranial PACT.^[^ ^60^, ^66^ ^]^ Third, the fundamental limitations of PACT (e.g., its limited bandwidth) remain. With additional research, a deep learning‐based approach can overcome this problem.^[^ ^67^, ^68^, ^69^ ^]^

In conclusion, our study successfully demonstrated that a whole‐brain PACT system can comprehensively monitor early‐stage ischemic stroke in vivo. The high‐definition whole‐brain PACT system offers a powerful tool for non‐invasive, real‐time in vivo monitoring of cerebrovascular changes, facilitating early diagnosis, enabling effective management, and guiding therapeutic decisions.

## 4 Experimental Section

### Multiparametric 3D Whole‐Brain PACT System

The system consisted of a 1024 element hemispherical ultrasound transducer array (center frequency 2.02 MHz; bandwidth 54%), a tunable optical parametric oscillator (OPO) laser (PhotoSonus M‐20, Ekspla, Inc, Lithuania), and a data acquisition (DAQ) system (Vantage 256, Verasonics, Inc., USA). A customized fiber bundle (Opotek, Inc., USA) was utilized inserted through the center of the transducer array to deliver pulse laser illumination to the target (20 Hz pulse repetition rate, 3–5 ns pulse duration, 690–1064 nm wavelength). The PA waves generated from the target were detected through a hemispherical transducer array, and the data were processed by the DAQ system. The data acquisition process was synchronized with the output trigger of the laser. The signals collected from the 1024 channels were reconstructed into a 3D single volume photoacoustic (PA) image, using the phase‐rotation beamforming algorithm with CUDA. To obtain whole‐brain images, a combination of raster scanning and rotary scanning was employed. First, raster scanning was conducted using a 3‐axis gantry system (LSQ150A, Zaber, Inc., USA) at a specific angle position (raster scanning in XY direction in Figure 1a). Then, after the rotary scanner was rotated by 30 degrees around the x‐axis (Figure 1a), raster scanning was performed again. During the rotation, the motor fixed and turned the entire body of the rat (Figure S6, Supporting Information). To prevent the brain from moving, a customized brain holder was used to secure it in a place. These processes were repeated for three angles to achieve whole‐brain image. Finally, the volumes obtained from each angle were compounded together to acquire a 3D whole‐brain image. Calibration was performed through phantom imaging to compound the rotated volumes (Figure S7, Supporting Information. For precision, data was acquired at five different angles, each separated by 12.5 degrees. The rotational coordinates were determined using 2‐thread‐phantom images. Based on these coordinates, image compounding was performed through rotational and translational transformations, which were further validated through leaf imaging. As a result, the expansion of the FOV was confirmed by employing the partially‐rotating module in two types of phantom imaging. For acoustic matching, the rat was positioned half‐submerged in a water tank affixed to the optical table. A custom‐designed mask was connected to an anesthesia system (VIP3000 Veterinary Vaporizer, Midmark, USA) to continuously anesthetize the rat with isoflurane throughout the experiment. A water circulator (C‐WBL, Changsin Science, Republic of Korea) regulated the water temperature, keeping the rat's body temperature stable. After all in vivo experiments, rats were sacrificed in accordance with guidelines approved by Pohang University of Science and Technology.

The HbT, SO_2_, and vessel images used in the analysis of this study were created through the following process: First, spectral unmixing was performed on the images obtained from six wavelengths (756, 780, 796, 820, 866, 900 nm) to calculate the concentrations of oxy‐hemoglobin (HbO) and deoxy‐hemoglobin (HbR). Before spectral unmixing, 3D median filter and volume smoothing were applied. Additionally, each wavelength image was compensated for the measured energy of the corresponding laser wavelength (ES220C, Thorlabs, Inc., USA), and the optical fluence was also compensated for each wavelength based on Beer‐Lambert's law, assuming an exponential curve.^[^ ^70^ ^]^ Subsequently, spectral unmixing was conducted using pixel‐based linear least squares (LSQ) fitting.^[^ ^25^ ^]^ Following that, the HbT concentration was calculated by summing the concentrations of HbO and HbR, and derived the SO_2_ values from the ratio of HbO to HbT. The 3D rendered images and movies displayed in this study were generated using 3D PHOVIS.^[^ ^71^ ^]^ Brain angiograms were created by applying the 3D Frangi vesselness filter^[^ ^72^ ^]^ to the HbT volume. The 3D Frangi vesselness filter is based on the Hessian matrix, which is widely used for PA microscopy and mesoscopy, can enhance vessels by exploiting the tubular structure.^[^ ^73^ ^]^ The Hessian function was applied in MATLAB with a sigma value of 1.^[^ ^74^ ^]^ Please see Figure S8 (Supporting Information) for a comparison of the images before and after applying the Frangi filter.

### Ex Vivo Skull Sample Imaging

Rats were anesthetized with 3% isoflurane with oxygen. The abdomen was incised and the heart was exposed to begin perfusion with saline through the left ventricle, during which the right atrium was incised to remove any residual blood. After perfusion, the rat's head was separated and the skull was harvested. To remove residual blood and air from the *ex‐vivo* skull, the isolated skull was immersed in water and held for 2 h.^[^ ^60^ ^]^ Finally, to confirm the skull's aberrations, the skull was positioned between two threads and the optical fiber output (see Figure S2a, Supporting Information). The image was acquired as a single volume without scanning, using a wavelength of 800 nm.

### Photothrombotic Stroke Model

Photothrombotic stroke was induced in four‐week‐old female Sprague Dawley (SD) rats weighing 250 grams. The rats were initially anesthetized with 3% isoflurane with oxygen, then the isoflurane concentration was reduced to 1.5% during surgery. During the surgical procedure, each rat's body temperature was maintained at 36.5–37 °C using a heating pad and temperature control system (Thermostar Homeothermic Monitoring System, RWD Life Science). To induce photothrombotic ischemia, rose bengal stain (15 mg kg^−1^, Sigma) was intraperitoneally injected 15 min prior to illumination. A midline incision was made in the scalp to expose the skull. A plate with a 5 mm diameter hole was placed 2.5 mm lateral and 2.5 mm posterior to the bregma region of the brain, exposing that area while blocking light from reaching the surrounding regions. A halogen illuminator (FOK‐150 W, Korea Optical Communication Co., Ltd.) was then used to expose the brain region within the hole in the plate to cold light, creating a photothrombus. The total light exposure time for photothrombus generation was 20 min. After stroke induction, the scalp skin was sutured with 6/0 nylon sutures (ETHILON nylon suture, ETHICON). After surgery, the rat was allowed to recover in a room‐temperature recovery room and observed for 24 h.

### Histological Analysis

The brain was collected after cardiac perfusion with saline, and tissue fixation was performed in 4% PFA for 24 h. After that, the tissue was stored in 30% sucrose until it sank to the bottom. The samples were removed, embedded in OCT (Tissue‐Tek O.C.T. Compound, SAKURA), frozen to −80 °C, and cut into 10 µm thick cryosections using a cryostat (CM1860, Leica). The sections were dried at RT for 30 min, blocked with blocking solution for 1 hour, and incubated overnight with primary antibodies at 4 °C under the following concentrations: anti‐goat CD31(1:200, AF3628, R&D) and anti‐rabbit CD34 (1:200, BS‐2038R, Bioss). Next, the sections were washed twice with PBS for 5 min each. After that, sections were incubated with secondary antibodies using rabbit anti‐Goat IgG Alexa Fluor 488 (1:1000, A11078, Invitrogen) and goat anti‐Rabbit IgG Alexa Fluor 594 (1:1000, A11012, Invitrogen) for 1 hour at room temperature and rinsed three times with PBS for 5 min each. Finally, the slides were coated with VECTASHIELD Antifade Mounting Medium with DAPI (Vector Laboratories) and observed under a confocal microscope (Confocal LSM900 with Airyscan 2, Carl Zeiss) at an appropriate excitation wavelength. The confocal images were quantitatively analyzed using ImageJ (NIH). For each location in the brain, an equal‐sized ROI was specified on the ipsilateral and contralateral cortex, and quantified the positive signal region of the total ROI area in the image for each channel (%Area).

In addition, 2,3,5‐triphenyltetrazolium chloride (TTC) staining was performed to confirm the photothrombotic stroke. One day after stroke induction, brain tissue was harvested and sectioned into 1 mm slices on the brain matrix (Alto Acrylic 1 mm Rat Coronal, Roboz Sugical). The sectioned tissues were stained with 2% TTC staining solution (Sigma‐Aldrich) for 20 min at RT and observed.

### Statistical Analysis

Statistical significance was derived using a one‐tailed t‐test. Significance levels are indicated as ^*^ *p* < 0.05, ^**^ *p* < 0.01, or ^***^ *p* < 0.005. All experimental data are described as mean values ± standard errors. Statistical tests were conducted by MATLAB R2021a (MathWorks, USA).

### Quantification Methods for In Vivo Whole‐Brain PACT

Morphological analysis was conducted based on the HbT concentration volume. To analyze vascular changes, the Frangi vesselness filter was applied to the HbT volume, obtaining a 3D whole‐brain angiogram.^[^ ^72^ ^]^ Again, note that applying a vesselness filter to the bulky structure of the thrombosis distorts the image. Therefore, to visualize the PT‐stroke model in Figures 2 and 4, the HbT volume image was used prior to applying the filter. However, the calculation of vessel density was performed on the angiogram obtained after applying the vesselness filter to the 3D ROIs excluding the thrombosis (as described immediately after the manual 3D ROIs designation). Additionally, Movie S4 (Supporting Information) shows the Day 1 angiogram image of the PT‐stroke model with the vesselness filter applied after the thrombosis disappeared. To calculate vessel density changes, the volume of interest was manually segmented from the brain volume. First, polygonal regions‐of‐interest (ROIs) were designated on the XY‐MAP image and then segmented the ROIs again on the YZ‐MAP, creating 3D ROIs. In time‐point images with strong thrombosis signals, thrombosis signals were excluded when designating ROIs. The vessel density within the acquired 3D ROIs was calculated by dividing the number of pixels containing vessels by the total number of pixels in the volume of interest. SO_2_ values of the vessels were also calculated by the same method by manually segmenting the 3D volume of the vessels and computing the values within this segmented volume. The average oxygen saturation (SO_2_) value for all pixels within the segmented volume ROI was calculated using the representative SO_2_ values for each region. To validate the SO_2_ measurements of the system, images of a human forearm were acquired and analyzed using the system. Four sets of 3D ROIs were created for both the radial artery and radial vein. Representative SO_2_ values were computed using the same method and plotted accordingly.

## Conflict of Interest

The authors declare no conflict of interest.

## Supporting information



Supporting Information

Supplemental Movie 1

Supplemental Movie 2

Supplemental Movie 3

Supplemental Movie 4

Supplemental Movie 5

## Data Availability

The data that support the findings of this study are available from the corresponding author upon reasonable request.
