# Correction to “Non‐Invasive Photoacoustic Cerebrovascular Monitoring of Early‐Stage Ischemic Strokes In Vivo”

**DOI:** 10.1002/advs.202502992

**Published:** 2025-05-11

J. Kim, J. Y. Kweon, S. Choi, H. Jeon, M. Sung, R. Gao, C. Liu, C. Kim, Y. J. Ahn, Non‐Invasive Photoacoustic Cerebrovascular Monitoring of Early‐Stage Ischemic Strokes In Vivo. Adv. Sci. 2025, 12, 209361.

https://doi.org/10.1002/advs.202409361

It has come to our attention that there was an oversight in the authorship list. One of the contributors, Jinge Yang, made significant contributions during the early stages of the project, specifically in the initial system design. However, his authorship was inadvertently omitted due to a focus on subsequent experiments and paper drafting, which we deeply regret. Therefore, we would like to correct the list of authors:

**Original**: Jiwoong Kim, Joo Young Kweon, Seongwook Choi, Hyunseo Jeon, Minsik Sung, Rongkang Gao, Chengbo Liu, Chulhong Kim, Yong Joo Ahn

**Corrected**: Jiwoong Kim, Joo Young Kweon, Seongwook Choi, Hyunseo Jeon, Minsik Sung, Jinge Yang, Rongkang Gao, Chengbo Liu, Chulhong Kim, Yong Joo Ahn

Affiliation of Jinge Yang (jgyang@caltech.edu):

1. Departments of Convergence IT Engineering, Medical Science and Engineering, Electrical Engineering, and Mechanical Engineering, Pohang University of Science and Technology (POSTECH), Cheongam‐ro 77, Nam‐gu, Pohang, Gyeongbuk 37673 Republic of Korea

2. Andrew and Peggy Cherng Department of Medical Engineering and the Department of Electrical Engineering, Caltech Optical Imaging Laboratory, California Institute of Technology, Pasadena, CA 91125, USA

We apologize for this error.

