# Supplementary material for: Non‐Invasive Photoacoustic Cerebrovascular Monitoring of Early‐Stage Ischemic Strokes In Vivo
Source: Adv Sci (Weinh). 2024 Dec 4;12(4):2409361. doi: 10.1002/advs.202409361 (PMC11775540; doi:10.1002/advs.202409361)
Supplement: Supplementary file 1 — Supporting Information [file ADVS-12-2409361-s006.docx]

Supporting Information

**Non-invasive Photoacoustic Cerebrovascular Monitoring of Early-stage Ischemic Strokes *in Vivo***

Jiwoong Kim^1^†, Joo Young Kweon^1^†, Seongwook Choi^1^†, Hyunseo Jeon^1^, Minsik Sung^1^, Rongkang Gao^3^, Chengbo Liu^3^*, Chulhong Kim,^1,4^*, and Yong Joo Ahn^1, 2^*

**Table S1**. Summary of studies about ischemic stroke and PACT system using hemispherical transducer array. PACT, photoacoustic computed tomography; PT, photothrombosis; pMCAO, permanent middle cerebral artery occlusion; tMCAO, transient middle cerebral artery occlusion; UCAL, unilateral common carotid artery ligation; O, monitored; X, not monitored; N/A, not applicable.

| References | Detector type  (# of elements) | Target | Ischemic diseases | Collateral circulation / neovascularization | Oxygenation changes |
| --- | --- | --- | --- | --- | --- |
| PACT systems about ischemic stroke | | | | | |
| Our study | Hemispherical (1024) | Brain | PT | O | O |
| [1] | Linear (256) | Brain | pMCAO | X | O |
| [2] | Hemispherical (128) | Brain | PT  MCAO | X | O |
| [3] | Cylindrical (128) | Brain | tMCAO | X | O |
| [4] | N/A (64) | Brain | MCAO | X | O |
| [5] | Ring (512) | Brain | UCAL  MCAO | X | X |
| [6] | Linear (128) | Brain (piglet) | PT | X | O |
| PACT systems using a hemispherical transducer array | | | | | |
| [7] | Hemispherical (512) | Whole-body | N/A | N/A | N/A |
| [8] | Hemispherical (1024) | Whole-body | N/A | N/A | N/A |
| [9] | Hemispherical  (512) | Whole-body | N/A | N/A | N/A |


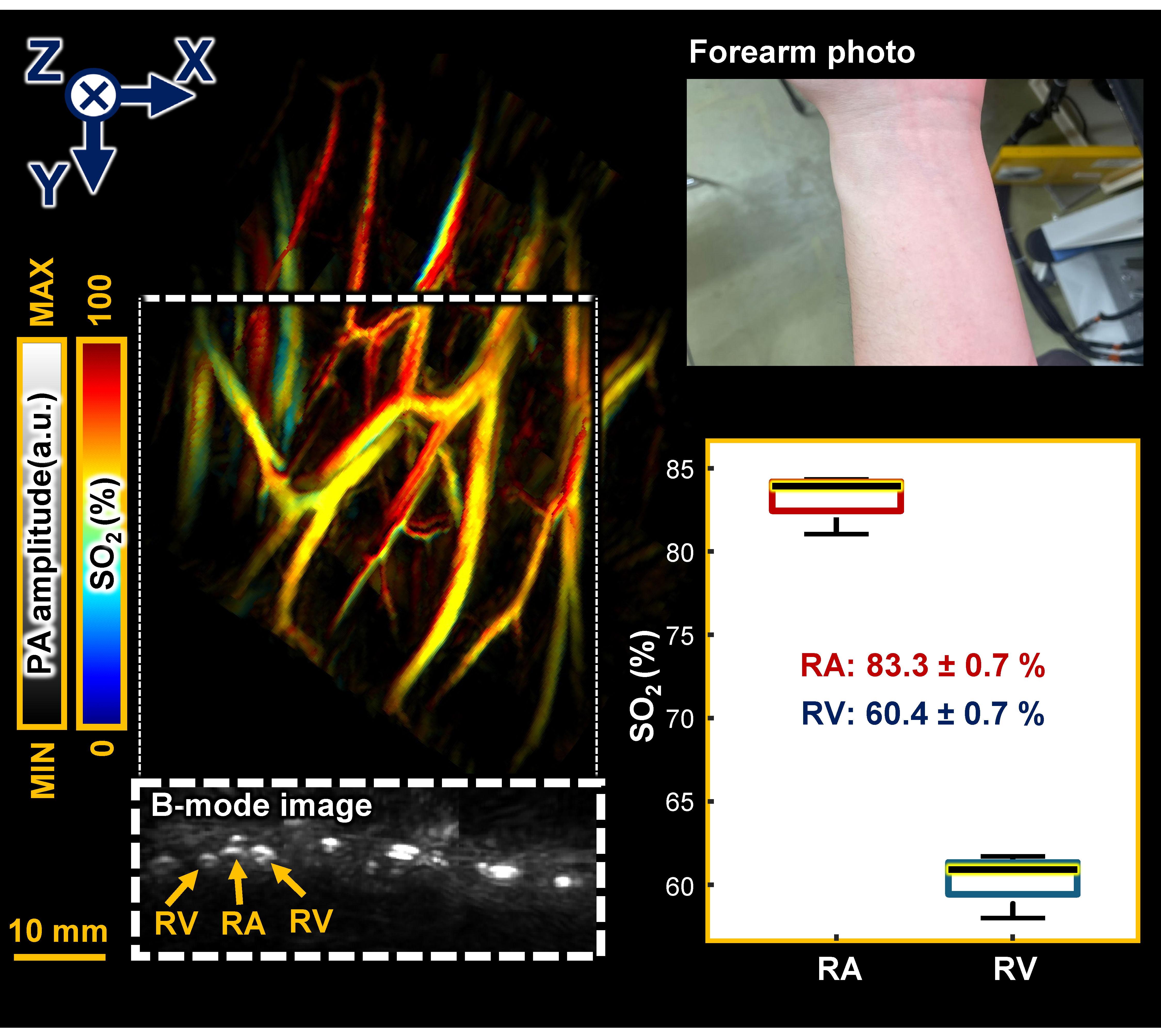


**Figure S1. Using the PACT system to image oxygen saturation in a human forearm.** An oxygen saturation labeled maximum amplitude projection image of a human forearm and a B-mode slice image. Quantification of the oxygen saturation in the radial artery and radial veins; RA, radial artery and RV, radial vein.

**
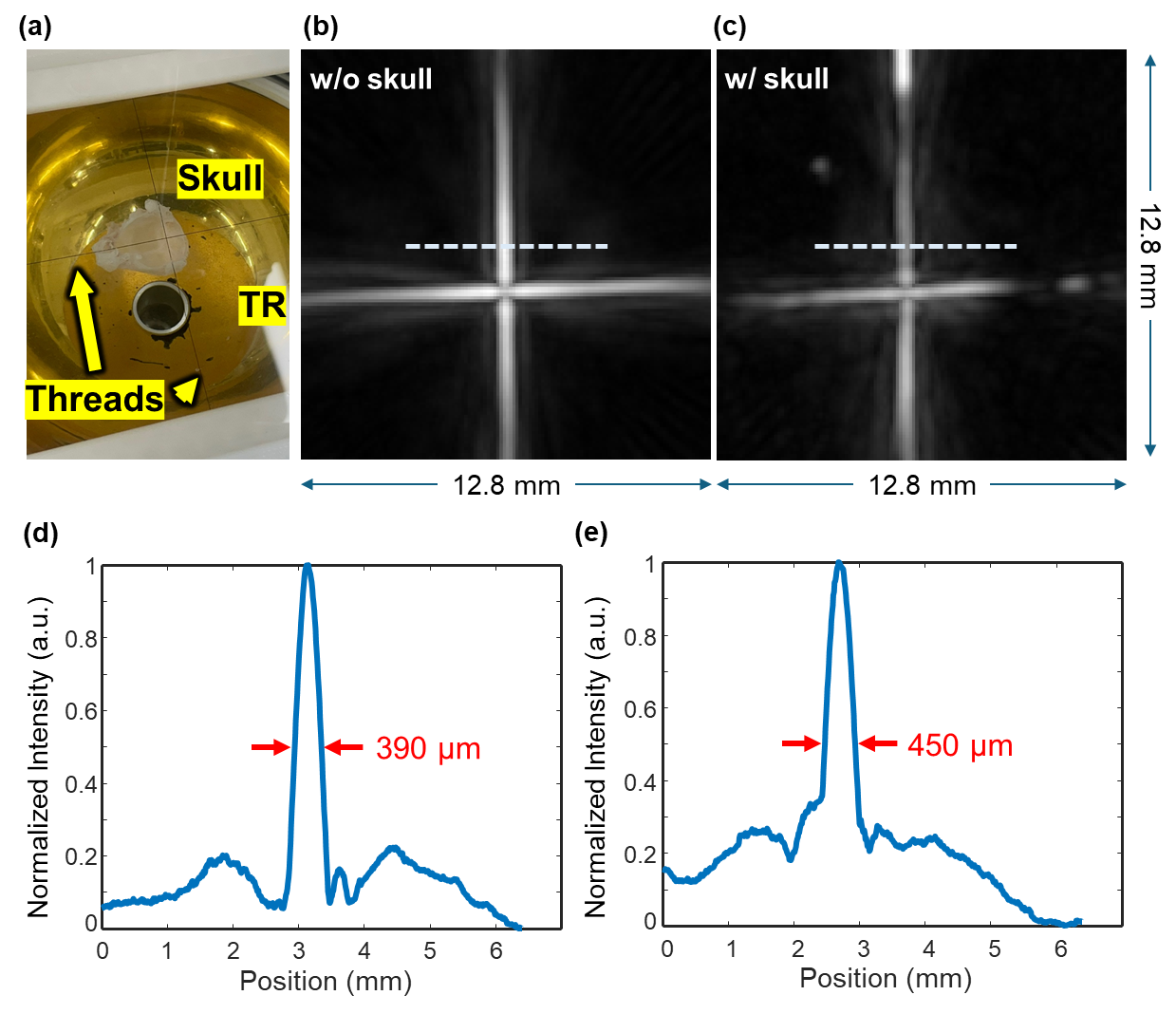
**

**Figure S2.** **Assessment of the resolution of the transcranial PACT system using a skull phantom.** (a) Photograph of the ex vivo skull sample. TR, transducer. Single volume PACT images of two threads (b) without and (c) with the skull sample. Normalized intensities of the profiles (white dashed lines): (d) without, and (e) with the skull sample.

**
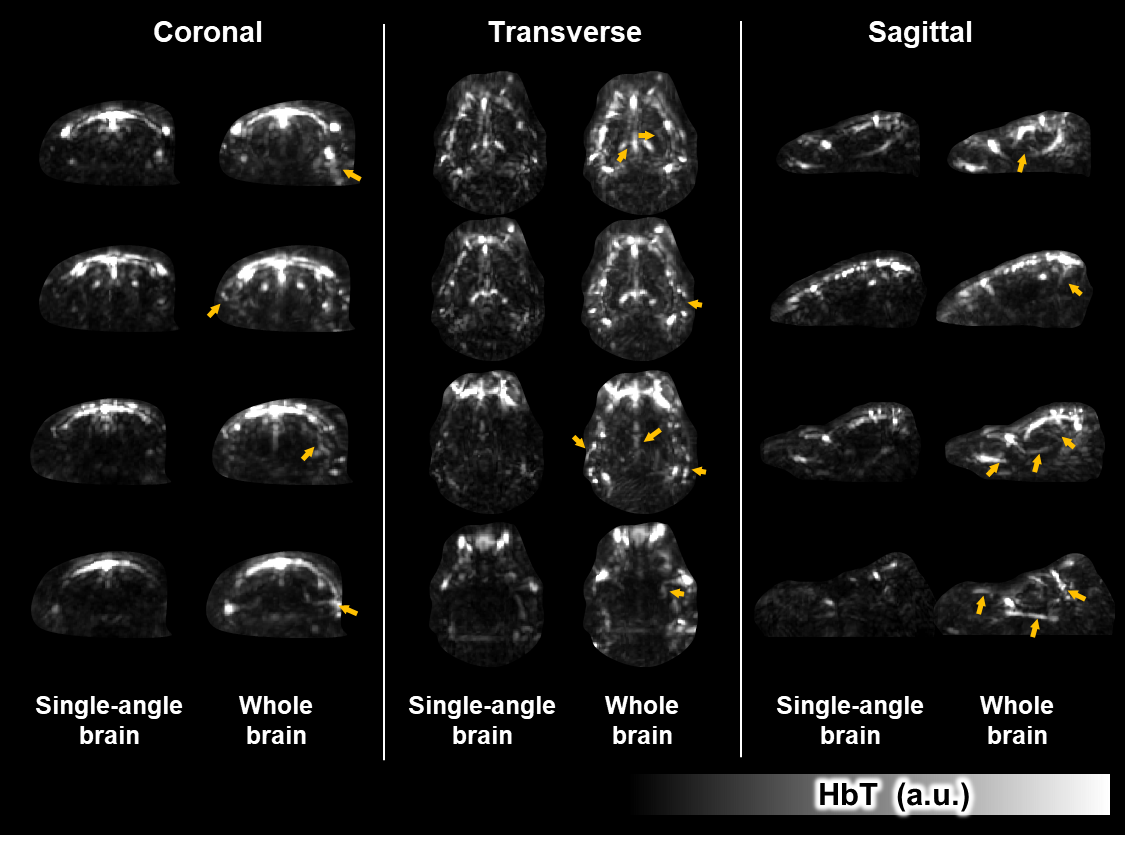
**

**F****igure S3.** **Comparison of 3D whole-brain PACT and single-angle brain images**. The orange arrows point to vessels that are clearly visible compared to single-angle.


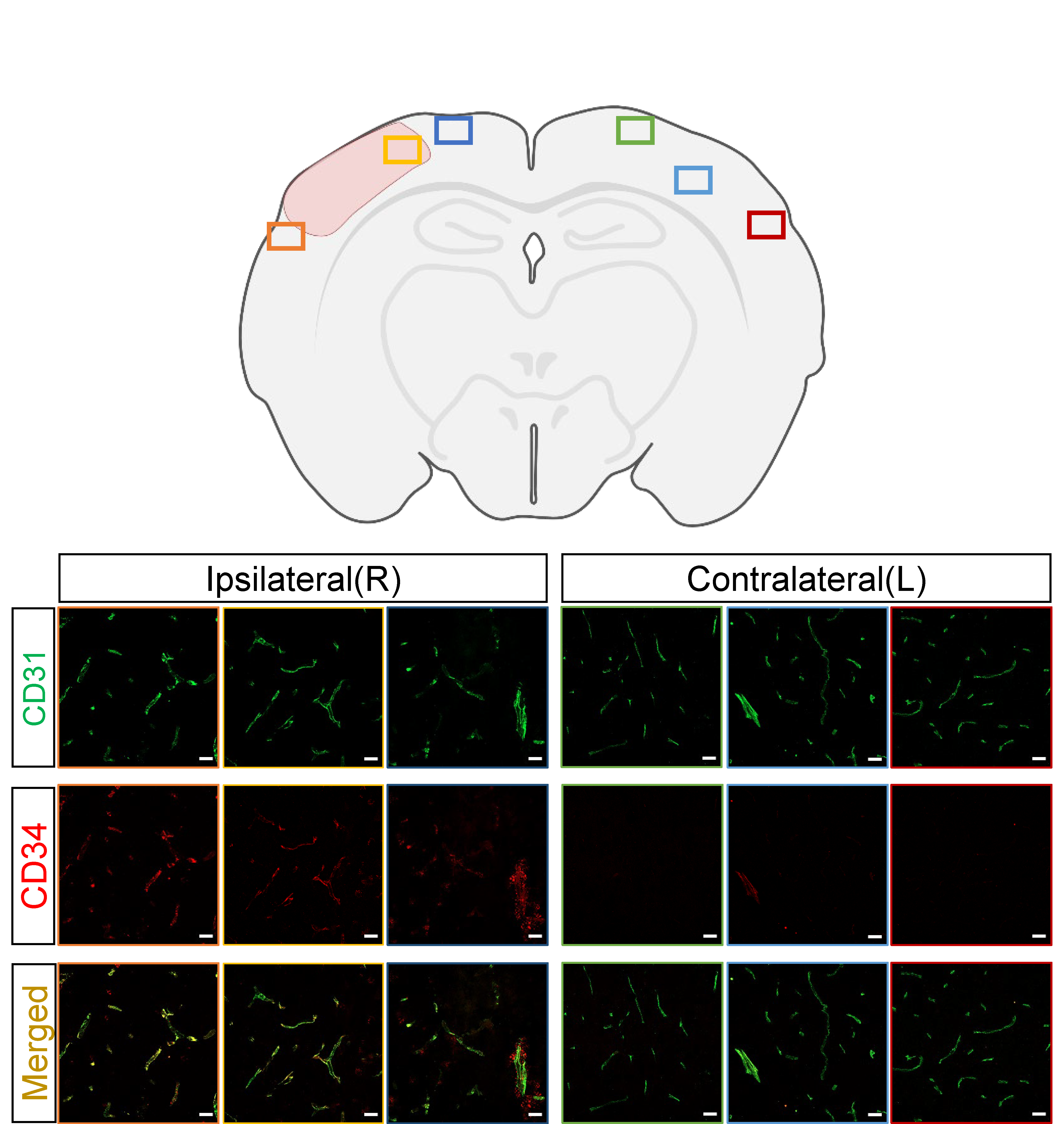


**Figure S4. Histological staining images from ipsilateral and contralateral hemispheres.**

**
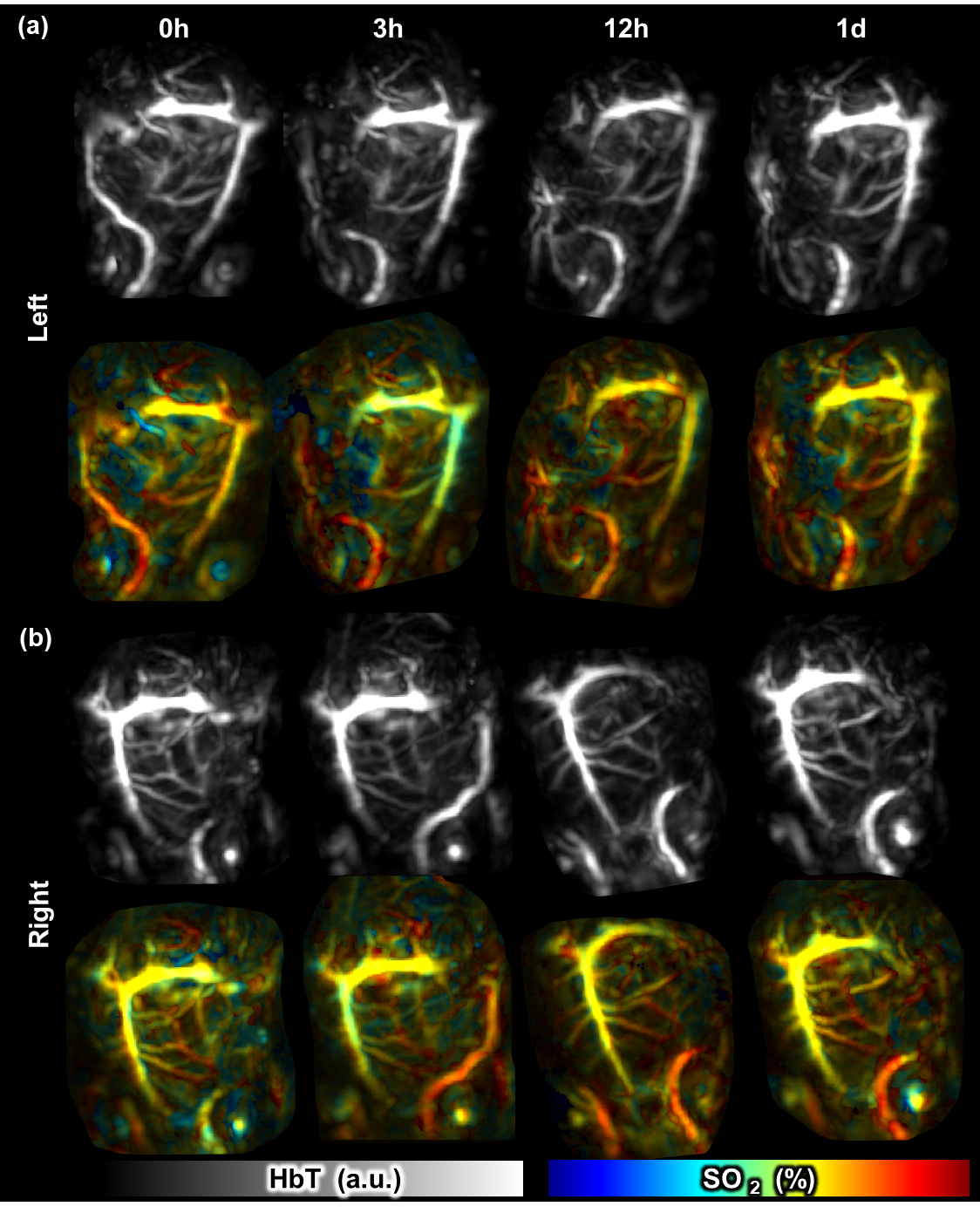
**

**Figure S5. Photoacoustic computed tomography of the control group, in which PT was not induced.** Maximum amplitude projection images and oxygen saturation distribution images at 0-, 3-, and 12- hours, and 1-day: (a) Left side and (b) Right side of the brain


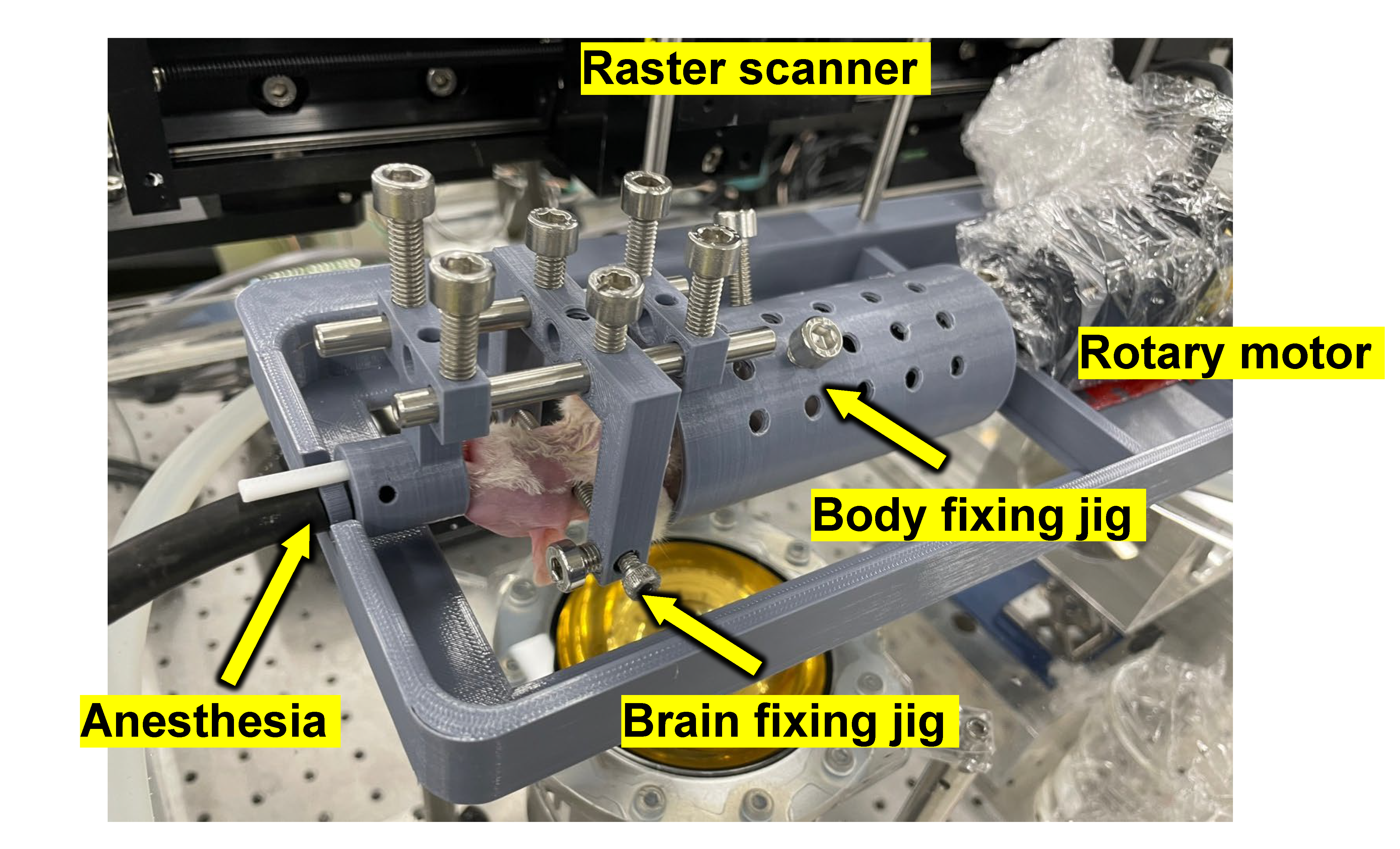


**Figure S6. Photograph of transcranial photoacoustic computed tomography system.**


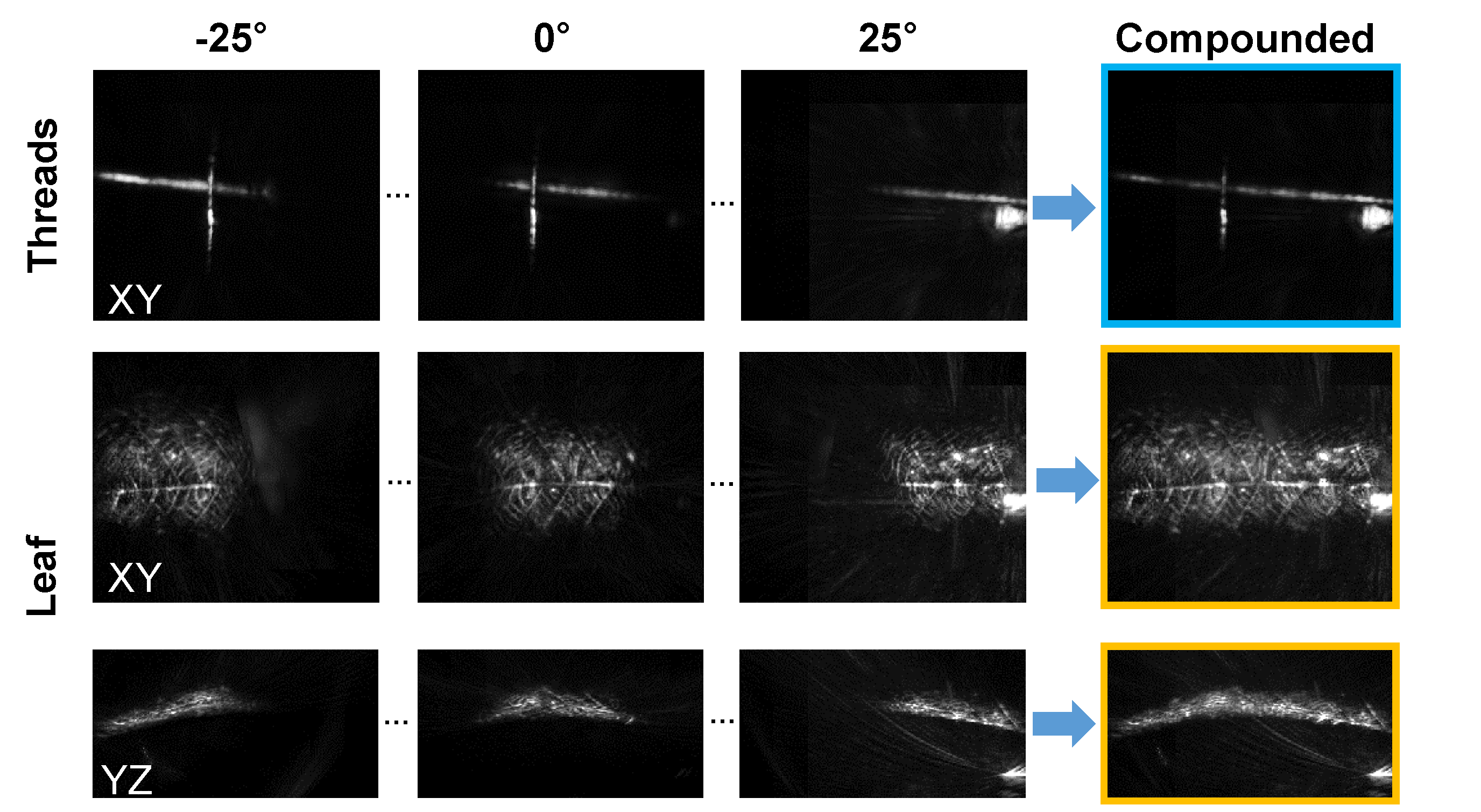


**Figure S7. Two-thread and leaf phantom images with raster and rotary scanning.**

**
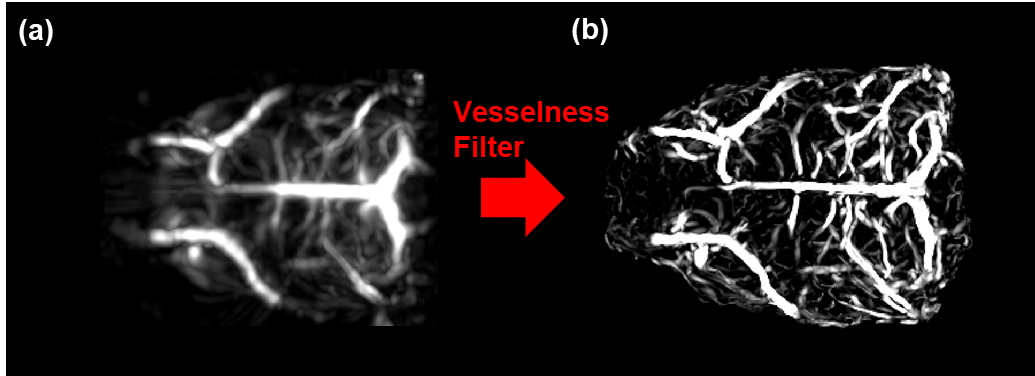
**

**Figure S8. Comparison of whole-brain images (a) before and (b) after applying the vesselness filter.**

**MOVIE LEGENDS**

Movie S1. Multiparametric 3D whole-brain PACT.

Movie S2. Coronal B-mode images of 3D whole-brain PACT.

Movie S3. Depth-encoded whole-brain PACT.

Movie S4. PACT-based angiogram at 1 day after stroke induction.

Movie S5. 3D whole-brain PACT images at pre-, 0-, and 6-hours, and 1-day after stroke induction.

**References**

[1] L. Menozzi, Á. Del Águila, T. Vu, C. Ma, W. Yang, J. Yao, *Photoacoustics*, **2023**, *29* 100444.

[2] J. Lv, S. Li, J. Zhang, F. Duan, Z. Wu, R. Chen, M. Chen, S. Huang, H. Ma, L. Nie, *Theranostics*, **2020**, *10* 816.

[3] R. Ni, M. Vaas, W. Ren, J. Klohs, *Neurophotonics*, **2018**, *5* 015005.

[4] M. Kneipp, J. Turner, S. Hambauer, S.M. Krieg, J. Lehmberg, U. Lindauer, D. Razansky, *PLOS ONE*, **2014**, *9* e96118.

[5] H. Li, Y. Zhu, N. Luo, C. Tian, *Journal of Biophotonics*, **2023**, *16* e202300235.

[6] J. Kang, X. Liu, S. Cao, S.R. Zeiler, E.M. Graham, E.M. Boctor, R.C. Koehler, *Journal of Neural Engineering*, **2022**, *18* 065001.

[7] S.K. Kalva, X.L. Deán-Ben, M. Reiss, D. Razansky, *Nature Protocols*, **2023**, *18* 2124-2142.

[8] S. Choi, J. Yang, S.Y. Lee, J. Kim, J. Lee, W.J. Kim, S. Lee, C. Kim, *Advanced Science*, **2023**, *10* 2202089.

[9] J. Yang, S. Choi, J. Kim, J. Lee, W.J. Kim, C. Kim, *Laser & Photonics Reviews*, *2024,* 2400672.
